# Nimesulide induced leukocytoclastic vasculitis and hepatitis: a case report

**DOI:** 10.1186/s40064-015-1081-9

**Published:** 2015-06-30

**Authors:** Prasanta Kumar Bhattacharya, Bhupen Barman, Aakash Roy, Md Jamil, Monaliza Lyngdoh, Jaya Mishra

**Affiliations:** Department of General Medicine, North Eastern Indira Gandhi Regional Institute of Health and Medical Sciences (NEIGRIHMS) Mawdiangdiang, Shillong, 793018 Meghalaya India; Department of Pathology, North Eastern Indira Gandhi Regional Institute of Health and Medical Sciences (NEIGRIHMS) Mawdiangdiang, Shillong, 793018 Meghalaya India

**Keywords:** Leukocytoclastic vasculitis, Nimesulide, Hepatitis, Adverse drug reaction, Pharmacovigilence

## Abstract

**Background:**

Nimesulide is a non-steroidal anti-inflammatory drug with antipyretic and analgesic properties, which is still used in many countries despite its known hepatotoxicity. Along with hepatotoxicity it has also been associated with several other Adverse Drug Reactions (ADRs) including leukocytoclastic vasculitis (LCV).

**Case description:**

A 38 year-old female presented with history of acute onset fever for which she took tablet nimesulide and paracetamol combination (100 mg Nimesulide + 500 mg paracetamol tablet), 1 tab three times daily for 4 days, following which she developed rash all over the body. She also had clinical and biochemical evidence of acute hepatitis. Histopathological examination of the skin rash documented the presence of LCV. She was managed symptomatically with anti-inflammatory and supportive therapy and was not further exposed to nimesulide.

**Discussion and evaluation:**

Our case demonstrates occurrence of acute hepatitis and LCV associated with nimesulide intake. The case meets the defining criteria for the diagnosis of LCV preceded by history of nimesulide intake. There was also clinical and biochemical evidence of hepato-cellular damage which supports the concurrent development of hepatitis along with the development of LCV following nimesulide use. To the best of our knowledge there is no previous published report of LCV and hepatitis occurring concurrently in the same patient following nimesulide intake. Nimesulide should be added to the list of agents associated with these serious adverse drug reactions.

**Conclusions:**

Nimesulide has been a contentious drug over many years. Under such evidence of serious ADRs the scientific community should consider ensuring strict pharmacovigilance with respect to its use especially in the developing countries where such monitoring systems are inadequate.

## Background

Nimesulide is a non-steroidal anti-inflammatory drug (NSAID) with antipyretic and analgesic properties. It is a selective cyclooxgenase-2 inhibitor and has been used in the treatment of a variety of inflammatory condition for last three decades in many countries of the world. Although it has been claimed to have a lower incidence of adverse gastro-intestinal effects, this has never been clearly demonstrated. On the contrary, numerous reports of Adverse Drug Reactions (ADR) have been attributable to nimesulide in the literature (WHO ADR Newsletter 1999). Some of the reported adverse effects include peripheral oedema, gastritis, stomatitis, necrotising fasciitis, Reye’s syndrome and coagulopathy with elevated liver enzymes and acute hepatitis.

Hypersensitivity vasculitis (HSV), which is usually represented histopathologically by leukocytoclastic vasculitis (LCV), is a term commonly used to denote a small vessel vasculitis (Lie 1994). Approximately 50% of cases of LCV are idiopathic with drugs, malignancies and connective tissue disorders accounting for a majority of the remaining cases (Tai et al. 2006). In a study from India it has been shown that drugs were the most common etiological factor associated with hypersensitivity vasculitis (HSV) of which the most commonly implicated were NSAIDs (Khetan et al. 2012). Few reports of nimesulide induced vasculitis have also been described in the literature (Polimeni et al. 2006).

Nimesulide has been a drug which has been subjected to controversies in the past. However, in spite of reports of serious ADR its usage continues to be in vogue in medical practice in many developing countries. Though the association of cutaneous reactions and of hepatitis with nimesulide use has been described separately, to the best of our knowledge, there is no report in the literature of the concurrent development of both hepatitis and vasculitis with the use of this molecule. With this background we report from India a case of hepatitis and LCV associated with the use of nimesulide.

## Case description

A 38 year old female presented with history of acute onset fever with body ache for which she took tablet nimesulide and paracetamol combination (100 mg Nimesulide + 500 mg paracetamol tablet), 1 tab three times daily for 4 days, following which she developed rash all over the body. She also complained of itching all over her body and of high colour urine. There was no history of oral or genital ulcerations, photosensitivity or joint pain. There was no history of allergy to drug including paracetamol. There was no history of intake of herbal or dietary supplements.

**Figure 1:**
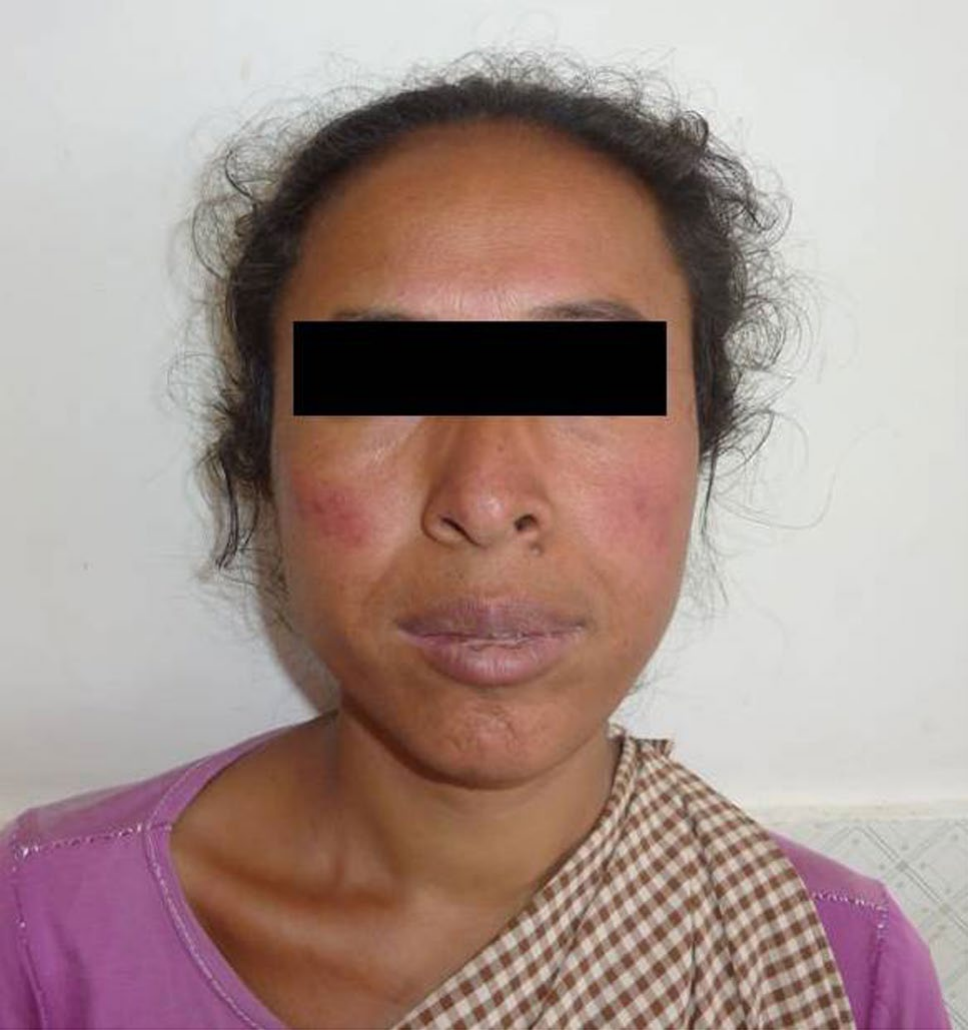
Showing the malar rash in the patient with leukocytoclastic vasculitis an hepatitis.

**Figure 2:**
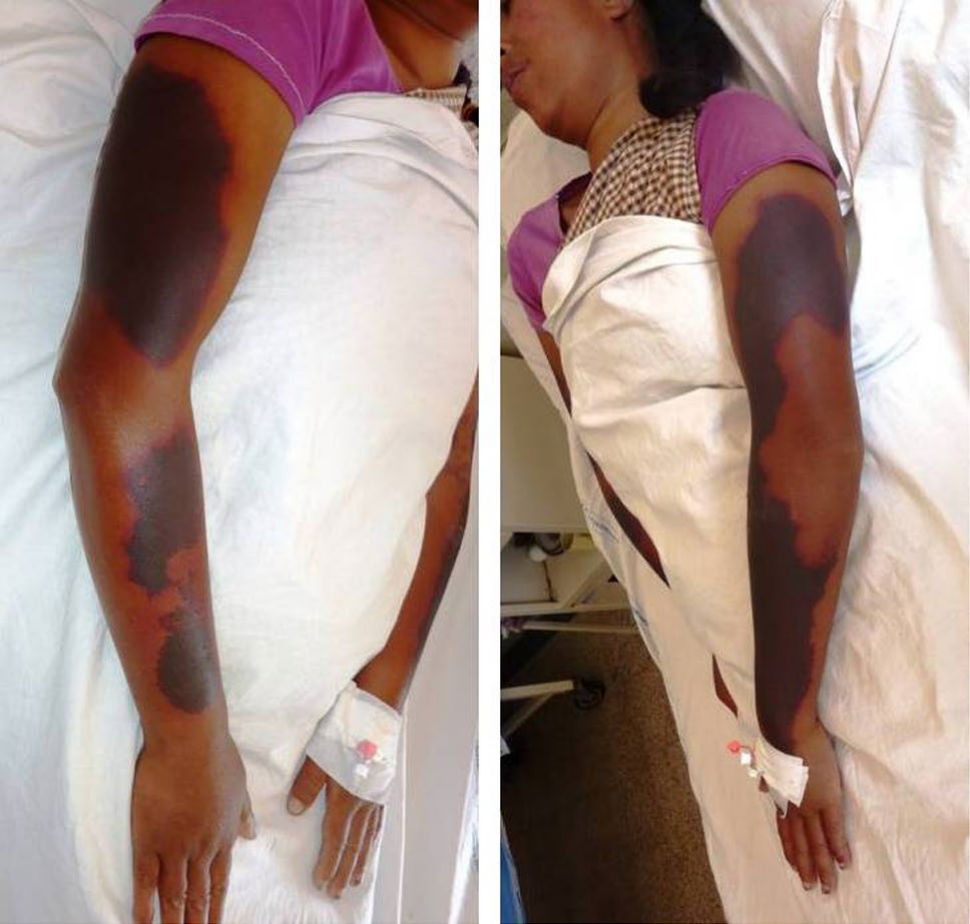
Showing the lesions in the upper limbs which have coalesced into large *blackish deep purple* patches.

**Figure 3:**
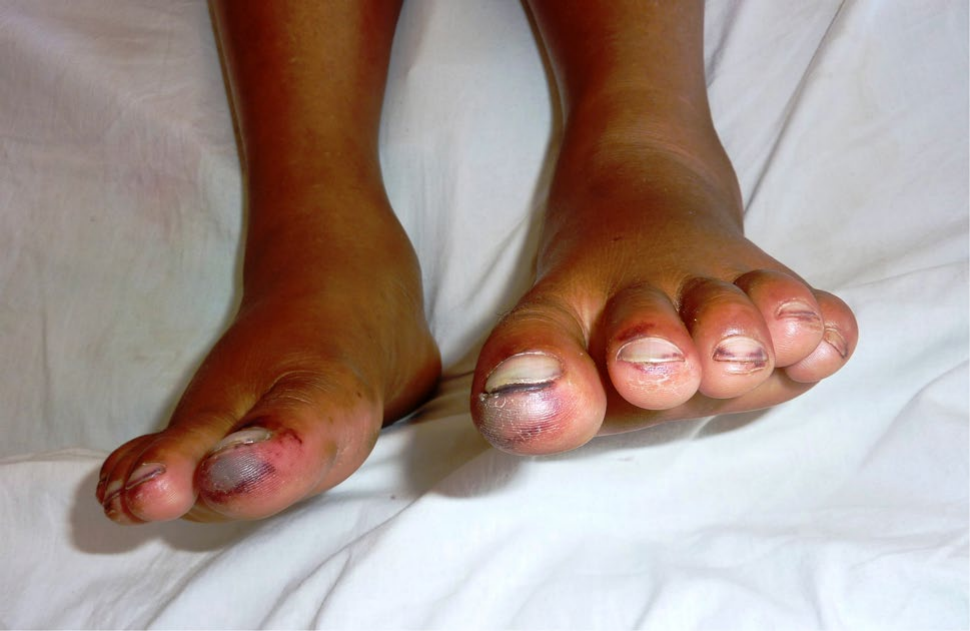
Showing the digital infarcts of the toes of both lower limbs.

**Figure 4:**
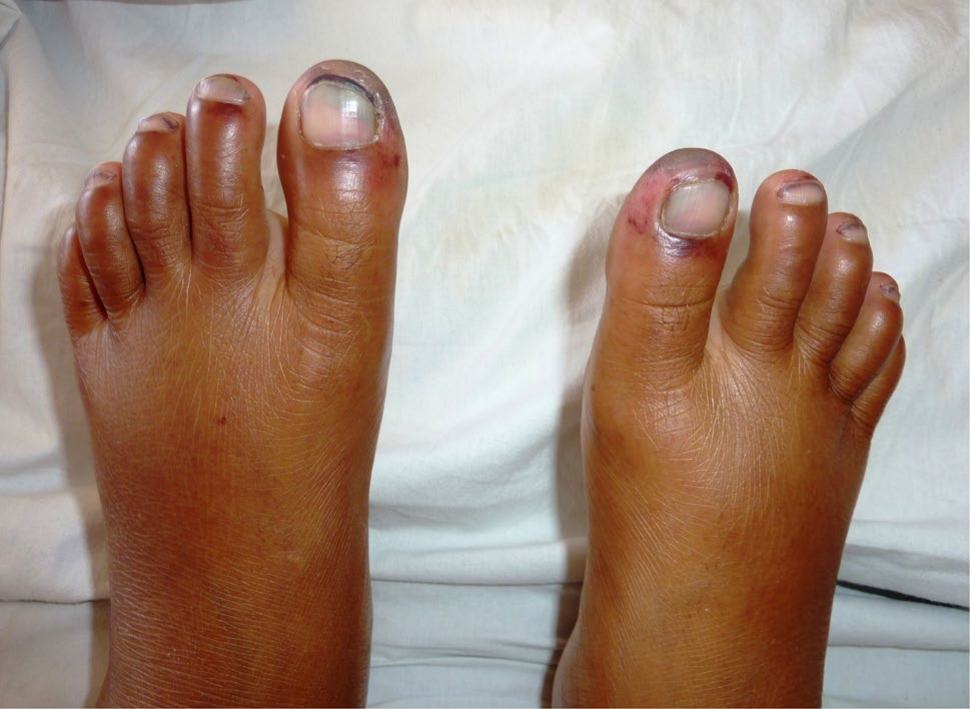
Showing the digital infarcts of the toes of both lower limbs.

**Figure 5:**
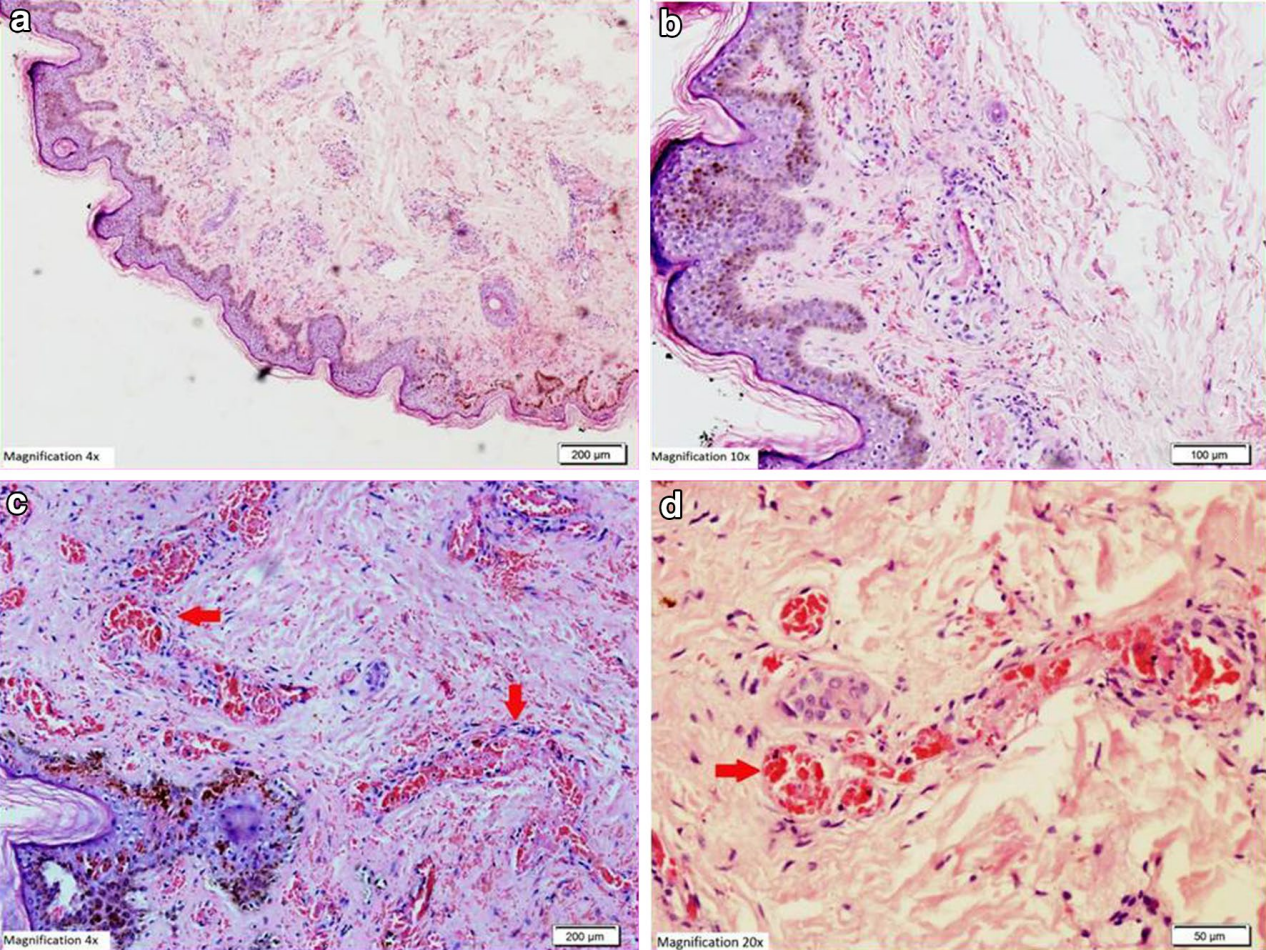
Histopathological examination of biopsy of the skin lesion from the upper arm showing leukocytoclastic reaction. **a**, **b** Hematoxylin and Eosin staining showing the epidermis lined by keratinizing stratified squamous epithelium with evidence of spongiosis. Most of the blood vessels in underlying dermis showing thrombus formation along with necrosis in the vascular wall and neutrophilic infiltration. **c**, **d** Periodic acid schiff staining of the same skin lesions showing amorphus PAS positive material deposits (*red arrows*) in form of intramural and intravascular thrombus.

**Figure 6:**
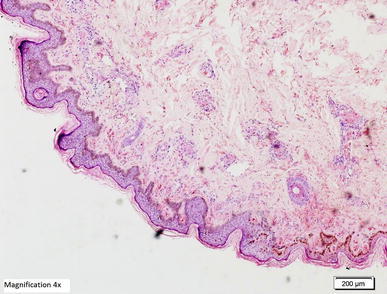
Hematoxylin and eosin staining showing the epidermis lined by keratinizing stratified squamous epithelium with evidence of spongiosis and neutrophilic infiltration (low magnification ×4).

**Figure 7:**
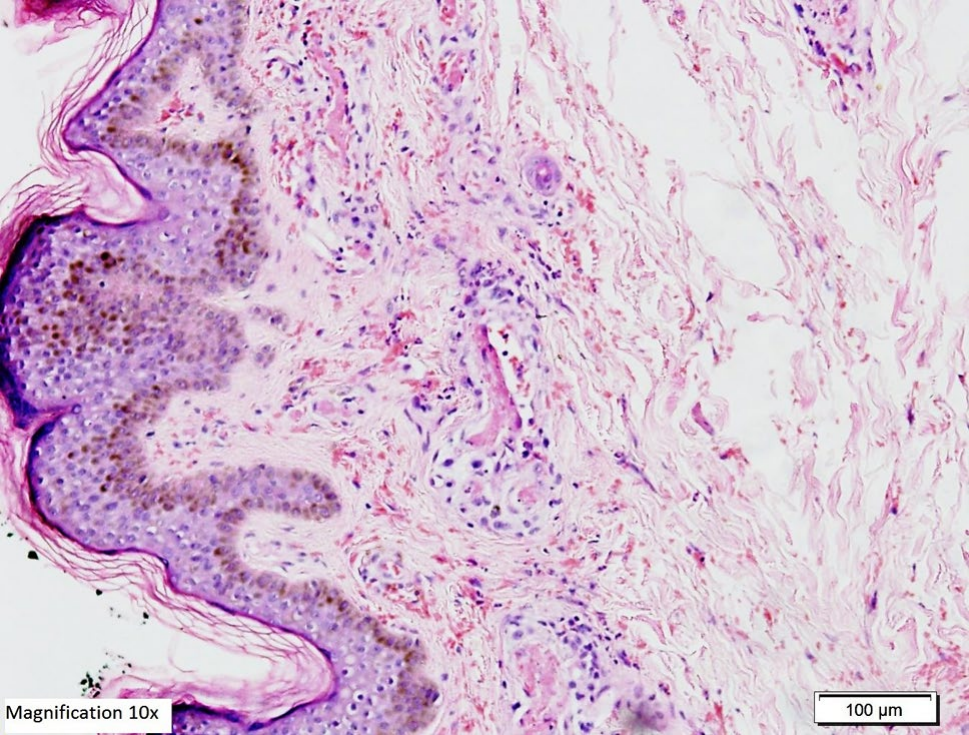
Hematoxylin and eosin staining showing the epidermis lined by keratinizing stratified squamous epithelium with evidence of spongiosis. Most of the blood vessels in underlying dermis showing thrombus formation along with necrosis in the vascular wall and neutrophilic infiltration (high magnification ×10).

**Figure 8:**
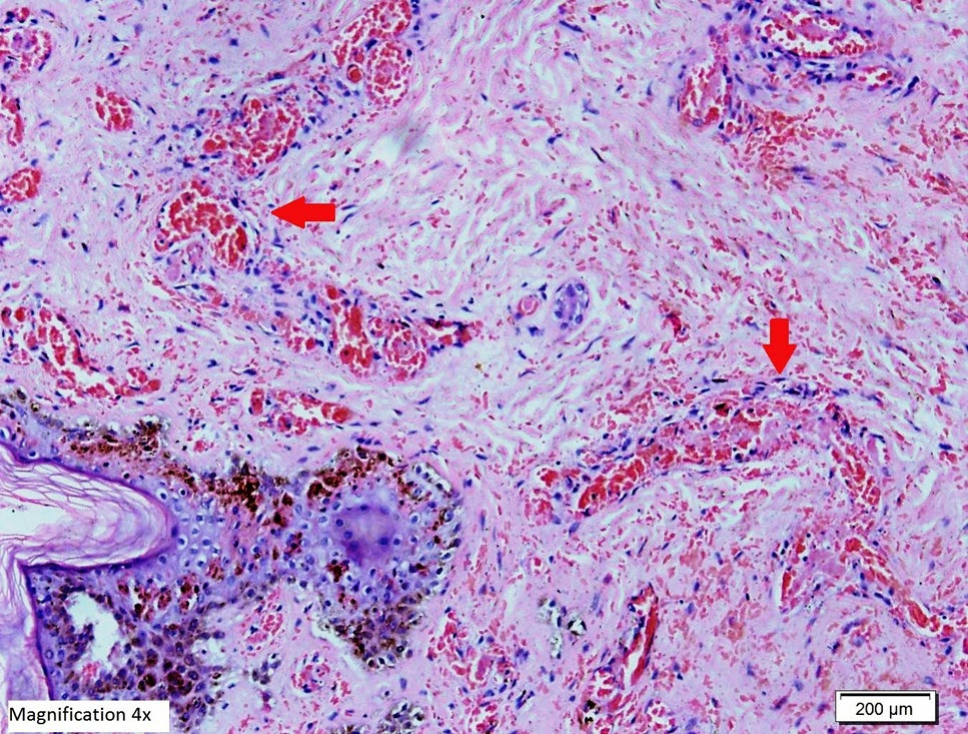
Periodic acid schiff staining of the same skin lesions showing amorphus PAS positive material deposits (*red arrows*) in form of intramural and intravascular thrombus (low magnification ×4).

**Figure 9:**
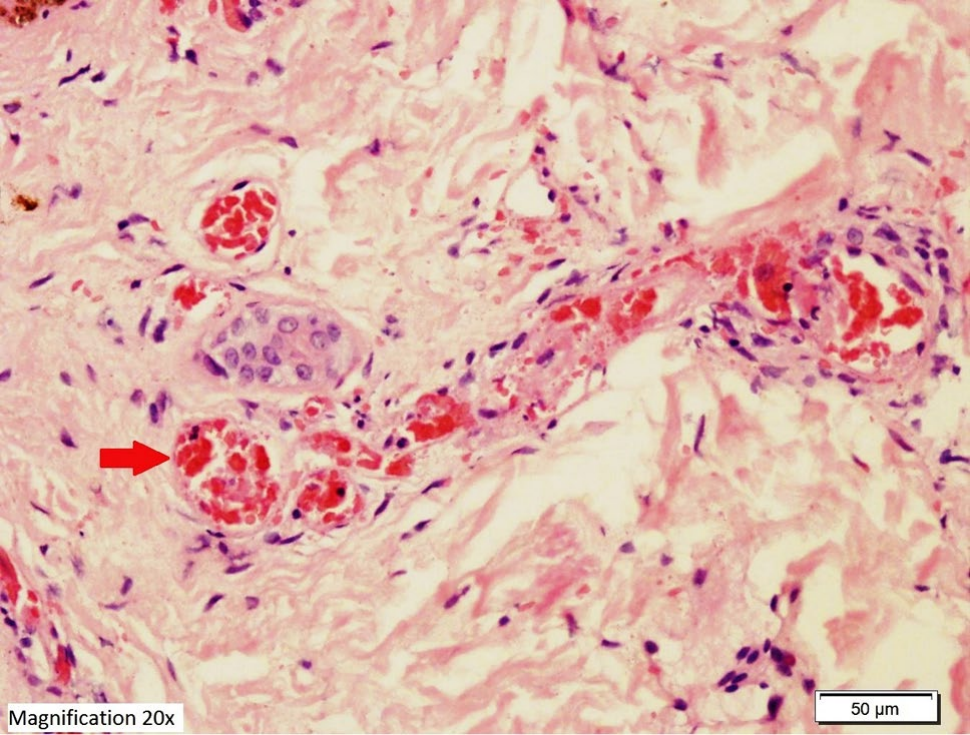
Periodic acid schiff staining of the same skin lesions showing amorphus PAS positive material deposits (*red arrow*) in form of intramural and intravascular thrombus (high magnification ×20).

On examination the patient was afebrile with a pulse rate of 76 beats per minute, blood pressure of 110/70 mmHg and respiratory rate of 16/minute. She had mild icterus, few small (<1 cm) non tender, right upper cervical lymph nodes and a soft and tenderly enlarged liver. Rashes were present over the trunk and all four extremities along with a malar rash over her face (Figure 1). Initially the rash was erythematous in the form of a palpable purpura and the lesions were small and punctate, but later on the lesions increased in size and coalesced to form extensive large blackish deep purple patches in both upper limbs (Figure 2). There were small digital infarcts of her toes bilaterally (Figures 3, 4). Other systems yielded no clinical abnormalities. Blood biochemistry (Table 1) revealed deranged liver function suggestive of cholestatic jaundice with mildly altered coagulation profile. Except for a mild anaemia the haematological profile was normal. Other biochemical parameters (blood glucose, serum electrolytes and renal functions) were normal. The anti nuclear antibody (ANA) and Rheumatoid Factor were negative. The viral markers for HIV-AIDS and hepatitis B & C were non reactive. Blood culture (three samples taken within 12 h of hospitalization), as well as urine culture were sterile. Except for hepatomegaly, the ultra-sonography of abdomen was normal. Arterial and venous Doppler studies of the extremities as well as CT angiography of upper limbs were also normal. Biopsy from the skin lesions of the upper limb showed features of LCV (Figures 5, 6, 7, 8, 9).

**Table 1:** Showing Laboratory findings in the patient with nimesulide induced hepatitis and leukocytoclastic vasculitis

Laboratory parameter (units)	Report		Reference values
	On admission	At discharge	
Haemogram			
Haemoglobin (gm/dL)	10.5	10.9	12–18
Total Leucocytic count (×10^3^/mm^3^)	8.50	8.60	4.0–11.0
Differential leucocytic count (%)			
Neutrophil	82	80	40–75
Lymphocyte	16	18	20–45
Monocyte	02	02	2–10
Eosinophil	00	00	1–6
Basophil	00	00	≤1
Platelet count (×10^3^/mm^3^)	200	Not done	150–400
Erythrocyte Sedimentation rate (mm/h)	02	Not done	0–20
Liver function tests			
Bilirubin (mg/dL)			
Total	6.0	1.9	0.3–1.3
Direct	3.4	0.5	0.1–0.4
Indirect	2.6	1.4	0.2–0.9
ALT (U/L)	173	126	7–41
AST (U/L)	295	42	12–38
Alkaline phosphatase (IU/L)	467	236	30–120
Protein, total (g/dL)	5.1	5.7	6.3–8.2
Albumin (g/dL)	2.6	3.0	3.5–5.0
Globulin (g/dL)	2.5	2.7	1.5–3.0
Coagulation profile			
Prothrombin time (s)	18.1	14.2	12.7–15.4
INR	1.47	1.38	1.34
APTT (s)	41.2	38.2	26.3–39.4
Renal profile			
Serum creatinine (mg/dL)	1.1	1.0	0.5–0.9
Blood urea (mg/dL)	42	38	10–50
Sodium (meq/L)	136	138	135–145
Potassium (meq/L)	3.6	3.8	3.5–5.5
Calcium (mg/dL)	9.2	9.3	8.7–10.2
Chloride (meq/L)	101	102	90–110
Blood glucose, random (mg/dL)	103	108	70–140
Blood culture	Sterile	Not done	
Urine culture	Sterile	Not done	
Antinuclear antibody	Negative	Not done	
Rheumatoid factor	Negative	Not done	
HIV I, II	Non Reactive	Not done	
HBsAg	Negative	Not done	
Anti HCV	Negative	Not done	

*ALT* alanine aminotransferase, *AST* aspartate aminotransferase, *INR* international normalised ratio, *APTT* activated partial thromboplastin time.

Considering the history of nimesulide intake preceding the development of hepatitis and LCV in the absence of any evidence of an infective or autoimmune etiology and normal Doppler and CT-angiography of the extremities, a diagnosis of nimesulide induced hepatitis and LCV has been made.

**Figure 10:**
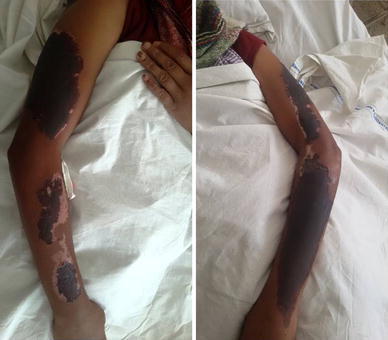
Showing improvement and gradual resolution of the skin lesions in the upper limbs of the patient.

The patient was treated with systemic corticosteroids (methyl prednisolone in a dose of 1 gm/day intravenously for 3 days followed by oral prednisolone in tapering dose) and other supportive measures. Gradually there was improvement with disappearance of the skin lesions (Figure 10) and healing of the digital infarcts. Blood parameters also showed improvement with normalisation of liver function tests by the ninth day (Table 1). She was discharged on the tenth day and was doing well on follow up after 3 weeks.

## Discussion and evaluation

In the present case report the patient had clinical and biochemical features of hepatitis which developed within 4–5 days of exposure to the drug. Liver biopsy was not performed. Drug induced hypersensitivity vasculitis can be identified on the basis of the following five characteristics (Calabrese et al. 1990): (1) age >16 years, (2) use of possible offending drug in temporal relation to the symptoms, (3) palpable purpura, (4) maculopapular rash and (5) biopsy of the skin showing neutrophils around an arteriole or venule. Our case also satisfies all the five criteria of LCV as evidenced clinically and histopathologically. Both the hepatitis and vasculitis of the patient gradually regressed and resolved following withdrawal of the offending drug and symptomatic treatment.

Nimesulide induced hepatotoxicity is a well known ADR. It has been shown to cause hepatic lesions more often in older women and the liver injury has been reported more commonly hepatocellular rather than cholestatic (Van Steenbergen et al. 1998). In another study, acute liver injury developed in 3 of 726 patients treated with nimesulide (McCormick et al. 1999). The exact molecular mechanism for hepatotoxicity has not been fully elucidated and in most instances it is thought to be an idiosyncratic reaction (Boelsterli 2002). Nimesulide induced hepatotoxicity has been shown to develop after about 15 days in two-thirds of patients (Bessone 2010), accordingly EMEA has recommended a dose of 100 mg per day (Bessone 2010). In our case, the patient had received 300 mg of nimesulide per day for 4 days, which could be the possible reason for the early development of hepatotoxicity. Because of its known toxicities, many developed countries have not allowed the manufacturing and marketing of nimesulide. Further, in some other countries its production and marketing was withdrawn after several instances of severe hepato-toxicity were reported following the use of nimesulide. In few cases this had even led to severe hepatic failure requiring liver transplantation or fatality (Polimeni et al. 2006). Despite these known toxicities it is still being used in some developing countries.

Hypersensitivity vasculitis (HSV), which is usually represented histopathologically by LCV, is a term commonly used to denote a small vessel vasculitis (Lie 1994). LCV or HSV may be idiopathic, drug induced or can occur be a part of a known disorder like infection. Many drugs including NSAIDs have been implicated in the development of LCV (Martinez-Taboada et al. 1997; Ekenstam and Callen 1984).

Although a precise pathophysiologic mechanism has not yet been identified for the development of LCV, several theories have been proposed. The involvement of T cells, which is evidenced by positive patch tests and lymphocyte transformation tests, has been frequently postulated (Britschgi et al. 2001).

The prognosis for drug induced LCV is generally good, with most patients undergoing spontaneous resolution within weeks to months (Rojeau 2005). Therapeutic modalities include identification of the offending drug and immediate discontinuation of drugs along with supportive management of the inflammatory response. There is increased morbidity and the potential for mortality if there is involvement of other major organ systems like kidneys, gastrointestinal tract, lungs, heart, or central nervous system.

## Conclusions

Nimesulide has been a contentious drug and due to its toxicities it has never been licensed for use in most developed nations of the world and has been withdrawn from others following reports of severe ADRs with this molecule. Unfortunately, the drug still continues to be used in many developing countries including India although its use in the pediatric population has been banned in India of late (The Gazette of India Extraordinary 2011). Under this scenario and keeping in mind the reports of ADR affecting more than one organ system as shown in our case study, the scientific community should emphasize the need for strict pharmacovigilance with the use of molecules like nimesulide, especially in the developing nations where such monitoring systems are inadequate. Further, as nimesulide administration is associated with acute liver failure, a strict enquiry about nimesulide intake should be made in any patient presenting with acute liver injury.

## Abbreviations

NSAID: non-steroidal anti-inflammatory drug
ADR: adverse drug reactions
LCV: leukocytoclastic vasculitis
ANA: anti nuclear antibody
HSV: hypersensitivity vasculitis.
